# Supplementary figures and images for: Integrated Analysis of Transcriptomic and Metabolomic Data Reveals the Mechanism by Which LED Light Irradiation Extends the Postharvest Quality of Pak-choi (Brassica campestris L. ssp. chinensis (L.) Makino var. communis Tsen et Lee)
Source: Biomolecules. 2020 Feb 7;10(2):252. doi: 10.3390/biom10020252 (PMC7072264; doi:10.3390/biom10020252)

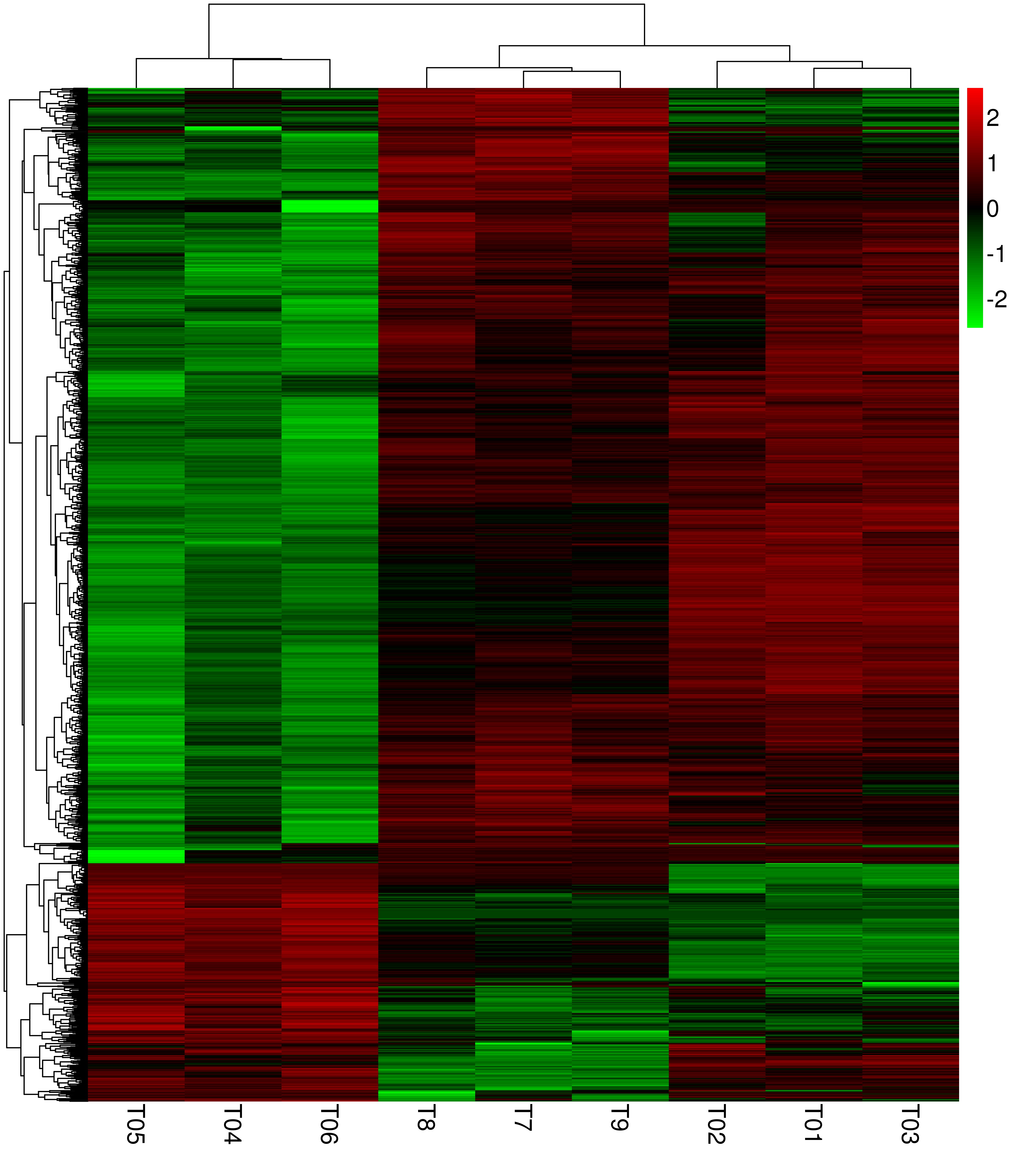

Supplement: Supplementary file 1 [file biomolecules-10-00252-s001.zip › Supplementary files/Supplementary Figure S1. The heatmap of differentially expressed genes identified in dark-stored control 5d (T04, T05, T06), LED treatment 5d (T07, T08, T09) (FC ≥ 2; FDR ≤ 0.01).jpg]
